# Molecular mechanisms of tungstate-induced pancreatic plasticity: a transcriptomics approach

**DOI:** 10.1186/1471-2164-10-406

**Published:** 2009-08-28

**Authors:** Jordi Altirriba, Albert Barbera, Héctor Del Zotto, Belen Nadal, Sandra Piquer, Alex Sánchez-Pla, Juan J Gagliardino, Ramon Gomis

**Affiliations:** 1 Diabetes and Obesity Laboratory, Endocrinology and Nutrition Unit, Institut d'Investigacions Biomèdiques August Pi i Sunyer (IDIBAPS), Hospital Clinic de Barcelona, Barcelona, Spain; 2 Centro de Investigación Biomédica en Red de Diabetes y Enfermedades Metabólicas Asociadas (CIBERDEM), Spain; 3 Centro de Endocrinología Experimental y Aplicada (CENEXA), Universidad Nacional de La Plata – Consejo Nacional de Investigaciones Científicas y Técnicas (UNLP-CONICET), Facultad de Ciencias Médicas, UNLP, La Plata, Argentina; 4 Department of Statistics, University of Barcelona, Barcelona, Spain; 5 Statistics and Bioinformatics Unit, Institut de Recerca Hospital Universitari Vall d'Hebron, Barcelona, Spain

## Abstract

**Background:**

Sodium tungstate is known to be an effective anti-diabetic agent, able to increase beta cell mass in animal models of diabetes, although the molecular mechanisms of this treatment and the genes that control pancreas plasticity are yet to be identified. Using a transcriptomics approach, the aim of the study is to unravel the molecular mechanisms which participate in the recovery of exocrine and endocrine function of streptozotocin (STZ) diabetic rats treated with tungstate, determining the hyperglycemia contribution and the direct effect of tungstate.

**Results:**

Streptozotocin (STZ)-diabetic rats were treated orally with tungstate for five weeks. Treated (STZ)-diabetic rats showed a partial recovery of exocrine and endocrine function, with lower glycemia, increased insulinemia and amylasemia, and increased beta cell mass achieved by reducing beta cell apoptosis and raising beta cell proliferation. The microarray analysis of the pancreases led to the identification of three groups of differentially expressed genes: genes altered due to diabetes, genes restored by the treatment, and genes specifically induced by tungstate in the diabetic animals. The results were corroborated by quantitative PCR. A detailed description of the pathways involved in the pancreatic effects of tungstate is provided in this paper. Hyperglycemia contribution was studied in STZ-diabetic rats treated with phloridzin, and the direct effect of tungstate was determined in INS-1E cells treated with tungstate or serum from untreated or treated STZ-rats, observing that tungstate action in the pancreas takes places via hyperglycemia-independent pathways and via a combination of tungstate direct and indirect (through the serum profile modification) effects. Finally, the MAPK pathway was evaluated, observing that it has a key role in the tungstate-induced increase of beta cell proliferation as tungstate activates the mitogen-activated protein kinase (MAPK) pathway directly by increasing p42/p44 phosphorylation and indirectly by decreasing the expression of raf kinase inhibitor protein (Rkip), a negative modulator of the pathway.

**Conclusion:**

In conclusion, tungstate improves pancreatic function through a combination of hyperglycemia-independent pathways and through its own direct and indirect effects, whereas the MAPK pathway has a key role in the tungstate-induced increase of beta cell proliferation.

## Background

The endocrine pancreas is continually remodelled [1] in a dynamic process involving the death and regeneration of beta cells. Though several mechanisms have been implicated in adult beta cell maintenance and renewal [2], it has been demonstrated that the proliferation of differentiated beta cells is the major mechanism for the maintenance of adult beta cell mass [3]. Nevertheless, it has recently been shown that endogenous progenitors can participate in the increase in beta cell mass in adult mice [4].

Studies in several animal models of diabetes have shown sodium tungstate to be an effective anti-diabetic agent, able to reverse hyperglycemia [5-9]. Tungstate treatment normalizes liver glucose metabolism in streptozotocin (STZ) and Zucker Diabetic Fatty (ZDF) rats [5,7], improves beta cell function in neonatal streptozotocin (nSTZ) rats [6], and also decreases hypertriglyceridemia in ZDF-rats [7]. In nSTZ-rats, tungstate not only restores glucose-induced insulin secretion, but also completely regenerates beta cell mass, leading to normoglycemia in animals even after withdrawal of the treatment [9]. Sodium tungstate has also been shown to exert insulin-mimetic effects in isolated hepatocytes [10] and to increase insulin content and enhance insulin secretion in the presence of glucose and various segretagogues in an insulinoma cell line and the perfused pancreas [11,12].

The aim of this study is to unravel the molecular mechanisms of tungstate action in the pancreas. Streptozotocin is a diabetogenic compound and is known to specifically destroy beta cells in the pancreas, obtaining a diabetic animal model with impaired endocrine and exocrine function and reduced beta cell mass. In this model, we identified the pancreatic gene expression changes induced by the streptozotocin damage and the partial recovery of function after treatment with tungstate. The data presented here show that tungstate treatment modifies the expression of a set of genes which participate in the tungstate-induced pancreatic plasticity, with a recovery of exocrine function and a partial recovery of endocrine function and beta cell mass. Moreover, we demonstrate that most of these effects are independent of the recovery of hyperglycemia and due to direct and indirect effects of tungstate in the pancreas. Further study of these pathways may lead to the discovery of new strategies for modifying pancreatic plasticity.

## Results

### Effects of tungstate treatment on blood and beta cell parameters

**Figure 1:**
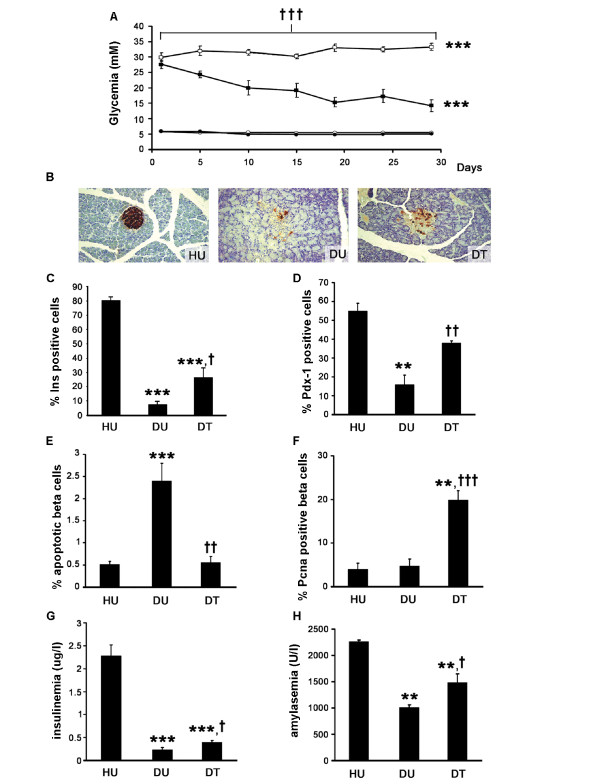
**Effects of tungstate administration on blood and beta cell parameters**. **a **Glycemia evolution of healthy untreated (open circles), healthy treated (filled circles), diabetic untreated (blank squares) and diabetic treated (filled squares) rats, n = 9. **b **Representative images of pancreatic sections stained with insulin (magnification 20×) for HU, DU and DT.**c, d **The percentage of insulin (**c**) and Pdx-1 (**d**) positive cells in the islets was analyzed from the pancreas (at three different levels), n = 6. **e, f **Beta cell apoptosis (**e**) and proliferation (**f**) were assessed by propidium iodide and Pcna staining, respectively, n = 8–10. **g, h **Insulin (**g**) and amylase (**h**) serum levels at the end of the tungstate treatment, n = 8–10. ***p < 0.001 and **p < 0.01 compared to healthy untreated animals, ^†††^*p *< 0.001, ^††^*p *< 0.01 and^†^*p *< 0.05 compared to diabetic untreated animals.

As previously described [5,8], administration of sodium tungstate significantly decreased glycemia in STZ-rats, while no changes were observed in the healthy animals (Figure 1a). Similarly, the administration of tungstate to STZ-rats also increased beta cell mass, though without reaching the levels found in the healthy animals (percentage of insulin area in the total pancreatic area, healthy untreated [HU]: 1.77 ± 0.27, healthy treated [HT]: 1.82 ± 0.26, diabetic untreated [DU]: 0.05 ± 0.01 p < 0.01 *vs. *HU, diabetic treated [DT]: 0.20 ± 0.05 p < 0.01 *vs. *both DU and HU, n = 6). In agreement with previous reports [8], tungstate treatment did not induce any significant modification in the healthy animals, and consequently, no further morphological analysis of these animals was performed. Figure 1b shows representative images of insulin-immunostained pancreas. Moreover, tungstate treatment in the diabetic animals also induced a significant increase in the number of positive insulin and Pdx-1 cells in the islets (Figures 1c and 1d). In order to understand the mechanisms involved in the increase in beta cell mass, we measured beta cell replication and apoptosis rates. As shown in Figure 1e, the diabetic rats had a higher number of apoptotic cells than the healthy animals. The treatment significantly decreased the rate of beta cell apoptosis, reaching levels comparable to those found in the healthy animals (Figure 1e). Moreover, tungstate increased beta cell replication in the diabetic animals (Figure 1f). These increases in beta cell mass and function were accompanied by a significant increase in blood insulin levels in the diabetic treated animals (Figure 1g). Therefore, the increased beta cell mass observed could be ascribed to a combination of the recovery of the apoptotic levels and the increased proliferation levels. This rise does not normalize beta cell mass, probably due to the extremely low levels of beta cell mass in the STZ-rats and the cells' limited capacity for recovery and replication. Nevertheless, the stimulation of the treatment permits a four-fold increase in beta cell mass and a two-fold increase in insulinemia in the animals. Moreover, although streptozotocin is a beta cell-specific toxin, it has also been reported that STZ-rats have impaired exocrine function [13], which was confirmed by the lower blood amylase levels in the diabetic animals; these levels were also improved by tungstate administration (Figure 1h).

### Gene expression profile of diabetic treated animals

**Figure 2:**
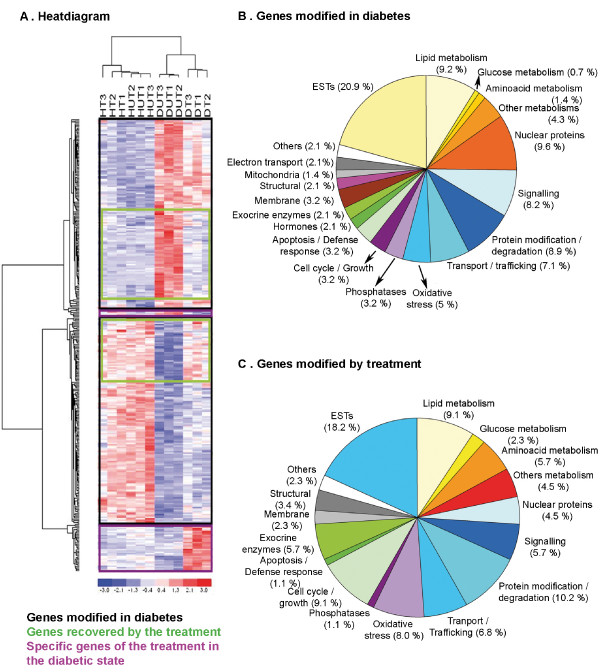
**Differential gene expression in the pancreas of the diabetic untreated and treated animals**. **a **Clustering of all the genes differentially expressed (horizontally) and the samples from the different experimental groups (vertically) using the dChip software.**b, c **Functional classification of the genes differentially expressed in the pancreas of diabetic animals (**b**) and in the pancreas of diabetic treated animals (**c**). The analysis of the arrays and identification of the differentially expressed genes was performed as described in Additional file 9. The functional classification of the genes was performed with the help of the NetAffx and GeneOntology databases.

To understand the underlying molecular mechanisms of tungstate action in the pancreas and to identify the tungstate target genes, we used microarrays to determine the gene expression in the four experimental groups. We performed microarrays of the whole pancreas in order to evaluate the possible signals coming from exocrine, ductal and endocrine tissue which might explain the phenotype observed. The differential expression analysis identified 370 differentially expressed genes, with clustered values (Figure 2a). The heat diagram clearly shows that the genes selected make it possible to distinguish between the healthy and diabetic groups, and between the treated and untreated diabetic samples. This distinction elegantly mimics the glycemic phenotype observed: the glycemia pattern and gene profile are similar in the treated and untreated healthy animals, but not in the treated and untreated diabetic animals. Moreover, the heat diagram grouped these genes in three different clusters: genes modified by diabetes, genes whose expression was restored by the treatment, and genes modified only in the diabetic treated animals.

In Figure 2b, the 282 genes differentially expressed due to diabetes are grouped according to their function [for a complete list, see Additional file 1]. The analysis of the arrays from DT animals identified 88 differentially expressed genes (see Figure 2c for functional clustering, and Additional file 2 for the complete list). The majority of these genes (70%) represented genes whose expression was restored, in varying degrees, by the tungstate treatment. Among these genes are the ones involved in metabolism, protein modification, oxidative stress, and so on, showing that the treatment acts on several pathways which lead to the recovery of pancreatic function. Only 28 of the 88 genes were differentially expressed in DT animals alone [see Additional file 3].

### Validation of the array results

**Figure 3:**
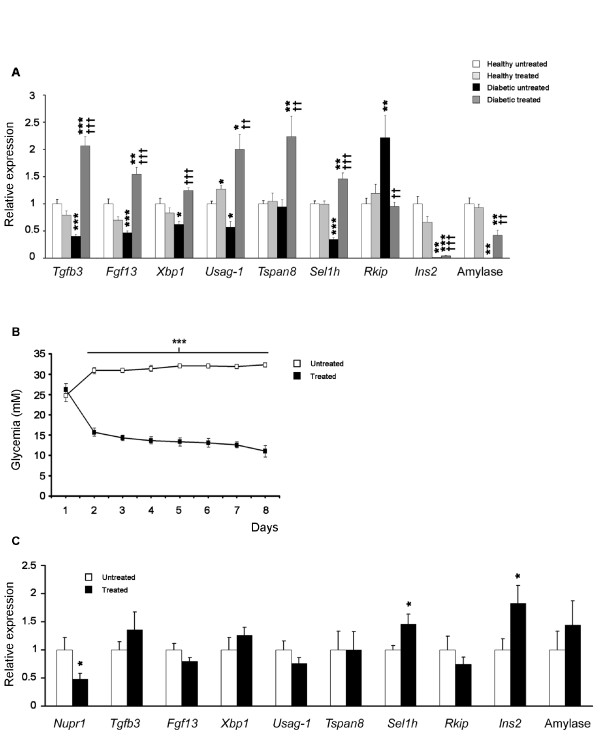
**Tungstate effects on the genes identified using the microarrays are specific and independent of glycemia normalization**. **a **From the list of specific genes modified in the diabetic treated animals, a significant number were chosen to corroborate the difference in expression levels detected by microarray analysis, using Real Time PCR, n = 7. ***p < 0.001, **p < 0.01 and *p < 0.05 compared to healthy untreated animals, ^†††^*p *< 0.001, ^††^*p *< 0.01 and ^†^*p *< 0.05 compared to diabetic untreated animals. **b **Diabetic animals were treated with phloridzin to decrease glycemia. Blood glucose levels during phloridzin administration, n = 12. ***p < 0.001 compared to vehicle treated animals. **c **mRNA expression levels from the pancreas of phloridzin treated animals were analyzed by Real Time PCR, n = 10. *p < 0.05 compared to vehicle treated animals.

After an exhaustive bibliographical research, from the list of all the genes modified by tungstate in the diabetic animals we chose a selection of genes whose function would help us to explain the effects observed in the pancreas of the tungstate-treated diabetic animals (further information available in Additional file 4 and Discussion), and we rechecked their expression using quantitative PCR. The genes chosen were transforming growth factor beta 3 (*Tgfb3*), fibroblast growth factor 13 (*Fgf13*), X-box binding protein 1 (*Xbp1*), uterine sensitization-associated gene-1 (*Usag-1*), tetraspanin 8 (*Tspn8*), suppressor of lin-12 1 homolog (*Sel1h*), and Raf kinase inhibitory protein (*Rkip*). The analysis is shown in Figure 3a. The results were similar to those found in the microarray analysis. Interestingly, gene expression was lower in DU than in HU animals (except *Tspn8*, which was unchanged, and *Rkip*, which was increased), whereas in DT animals, gene expression increased significantly as compared to DU, and sometimes it even surpassed HU. Insulin 2 and amylase were also included as controls. As in the microarrays and in the biochemical data, the expression of these genes was extremely low (almost zero) in the DU rats, and tungstate treatment partially restored amylase expression (a 45.98 ± 10.97 fold increase DT *vs. *DU, p < 0.01) and increased insulin expression (a 4.49 ± 1.10 fold increase DT *vs. *DU, p < 0.001).

### Tungstate effects are independent of glycemia normalization

Hyperglycemia has a critical impact on beta cell function [14] and gene expression [15]. To assess the involvement of the decrease in the hyperglycemia on tungstate effects, we decided to explore whether the gene expression changes induced by the treatment were just the consequence of the decrease in glycemia observed in the diabetic treated animals. To do so, diabetic rats were treated with phloridzin, which selectively inhibits active glucose reabsorption in the kidneys and, as opposed to insulin administration, leads to a decrease in glycema without any direct effect on pancreas function. Phloridzin administration led to a significant decrease in glycemia, though healthy values were not attained; interestingly, the levels reached were quite similar to those observed in DT animals (Figures 3b and 1a). Analysis of the expression of the genes validated by Real Time PCR showed that most did not present significant differences between the phloridzin administrated animals and their control counterparts (Figure 3c). Nevertheless, *Sel1h *and insulin expression increased significantly (insulin: 1.82 ± 0.32 fold increase, *Sel1h*: 1.45 ± 0.18 fold increase, p < 0.05 *vs. *untreated animals), although this increase was lower than the one observed in the tungstate-treated diabetic rats (insulin: 4.49 ± 1.10 fold increase, *Sel1h*: 4.21 ± 0.31, p < 0.001 *vs. *DU). Nuclear protein 1 (*Nupr1*), which has been shown to be regulated by glucose levels [16], was included as a positive control, and, as expected, it was significantly reduced in phloridzin-treated animals. Therefore, we conclude that tungstate effects on gene expression are independent of the decrease in glycemia.

### Tungstate increases beta cell proliferation indirectly through the modification of the serum profile

**Figure 4:**
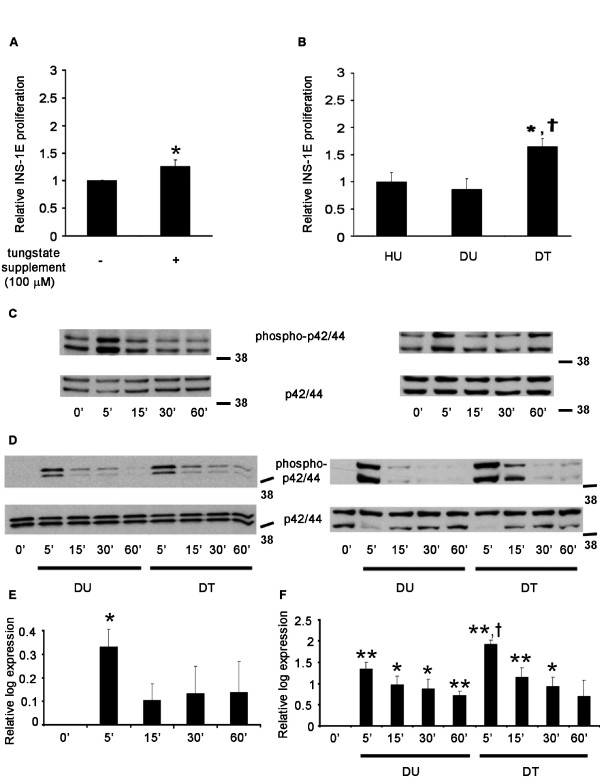
**Effects of tungstate and serum from tungstate treated animals on beta cell proliferation and analysis of p42/44 phosphorylation capacity**. **a **Tungstate induction of INS-1E cells proliferation. INS-1E cells were cultured with or without tungstate (100 μM) in the absence of fetal bovine serum. n = 5, *p < 0.05 *vs. *control. **b **Serum from diabetic treated animals enhances INS-1E proliferation. The cells were cultured in RPMI media containing 10% of the serum from the animals of each experimental group (HU, DU and DT). Serum samples were obtained after 5 weeks treatment with tungstate. n = 5. *p < 0.05 *vs. *healthy untreated animals and ^†^*p *< 0.05 compared to diabetic untreated animals. **c-f **INS-1E cells were treated (**c **and **e**) with tungstate, or with serum from the untreated and treated diabetic animals (**d **and **f**) for the minutes indicated. Lysates were immunoblotted (representative Western blots are shown, **c-d**), quantified (**e-f**) and expressed as the logarithmic fold change of phosphorylated p42/44 protein normalized by the total quantity of p42/44 for each time point *vs. *time 0 (n = 4–5). **p < 0.01 and *p < 0.05 *vs. *time 0 and ^†^*p *< 0.05 as compared to the same point time in the samples treated with serum from the diabetic untreated animals.

Tungstate administration to the diabetic animals increased beta cell proliferation. This increase was responsible, at least partially, for the beta cell mass recovery observed in the diabetic animals. Firstly, we assessed whether tungstate alone in culture was able to increase beta cell proliferation. As shown in Figure 4a, in culture, tungstate slightly increased INS-1E proliferation. However, this increase was much lower than the one observed *in vivo*; therefore, we wondered whether tungstate administered *in vivo *was able to exert a greater effect due either to the action of one of its metabolites or due to some indirect mechanisms. To shed some light on the matter, we cultured INS-1E cells in the presence of serum from the treated animals. Surprisingly, the sera from the diabetic treated rats were able to enhance INS-1E proliferation (Figure 4b). This increase was greater than the one observed when the cells were treated with tungstate directly. Moreover, when tungstate was added to a medium containing foetal bovine serum, no differences were observed (data not shown), suggesting that tungstate was not acting through the enhancement of some trophic factors present in the serum. In conclusion, tungstate acts on beta cell proliferation in diabetic animals probably through an indirect mechanism.

### MAPK pathway is involved in tungstate induced beta cell proliferation

Among several other cellular functions, the MAPK pathway plays a key role in cell replication [17]. One of the tungstate-specific genes that we have analyzed is Rkip, which is involved in the fine tuning of this important pathway in the islets through the inhibition of Raf-1 [18]. This gene was increased in the diabetic untreated animals compared to untreated healthy rats (inhibiting the MAPK p42/44 pathway), and the treatment returned its levels to normal (re-establishing MAPK p42/44 signalling) (Figure 3a). Moreover, previous studies [10] have demonstrated that sodium tungstate is able to activate the MAPKp42/44 pathway by increasing p42/44 phosphorylation in hepatocytes. Therefore, we decided to further evaluate the role of the MAPK pathway in tungstate-induced beta cell replication. We observed that tungstate was able to stimulate the MAPK pathway in INS-1E (Figure 4c and 4e). Similarly, the serum from the diabetic treated animals was also able to enhance the MAPK pathway more strongly than the serum from the untreated animals (notice the different scales used in Figures 4e and 4f); in addition, the activation of the pathway lasted longer (Figures 4d and 4f). This MAPKp42/44 activation pattern mimics the findings observed with the INS-1E proliferation in the presence of tungstate and serum from the different animals, showing also that serum from diabetic treated animals is a more potent proliferative agent and activator of the MAPK p42/44 pathway. Since it seemed that the stimulation of beta cell replication was linked to MAPK activation, we decided to culture INS-1E cells with PD98059, a MAPK/ERK kinase 1 (Mek1) inhibitor [19]. In the presence of PD98059, the serum from the treated animals induced a lower stimulation of beta cell replication (62% reduction, p < 0.05, n = 6), but no significant effect was seen in the serum from the untreated animals (8% reduction, p = n.s., n = 6). These data suggest that in order to exert its effects on beta cell replication, tungstate has to activate the MAPK pathway by both short-term (direct activation) and long-term mechanisms (normalization of *Rkip *expression), probably through an indirect mechanism involving either tungstate metabolization or the stimulation of some beta cell trophic factor(s).

## Discussion

This paper focuses on the molecular mechanisms by which tungstate administration modulates pancreatic plasticity in diabetic animals. We selected the streptozotocin rat model, which shows a high degree of pancreatic damage at both endocrine and exocrine levels [20]. In this situation, tungstate administration partially restores glycemia, insulinemia and amylasemia. Moreover, the morphological results clearly demonstrate that treatment increased beta cell mass in the pancreas and increased insulin and Pdx-1 positive cells in the islets. This increased beta cell mass can be ascribed, at least in part, to a combination of a decrease in beta cell apoptosis and an increase in islet proliferation.

In agreement with the metabolic phenotype, the pancreatic microarray analysis found no differences in the gene expression pattern between HU and HT animals, but it showed a very different pattern in DU animals as compared with both healthy samples. These changes are found not only in genes linked to endocrine function but also in those linked to exocrine function, probably due to the toxic effects of STZ. Finally, DT animals presented a specific pattern which was different from both DU animals and healthy animals, due to the effects of sodium tungstate on the diabetes background.

**Figure 5:**
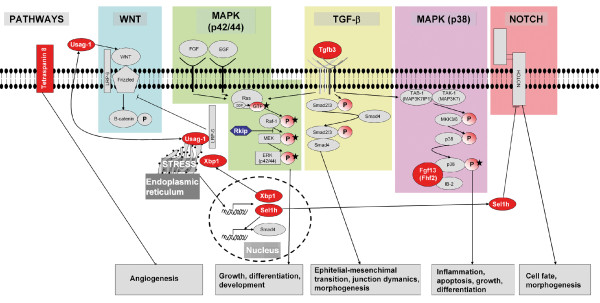
**Pathways involved in the effects of tungstate on the pancreas**. Microarrays from diabetic treated animals show that tungstate is acting through many interconnected pathways, which lead to an overall improvement of pancreas function. Some of the genes found are over-expressed (red) or diminished (blue) in diabetic treated animals and are involved in the control of several cellular pathways, either activating (arrows) or repressing (lines with a perpendicular line at the end). The final effects of the activation or inhibition of these pathways are described at the bottom of the figure. Moreover, the phosphorylations are represented as circles with a P inside, and the tungstate effects described for enhancing the phosphorylation of different key proteins are shown with a black star [9,10,25].

The analysis of the microarray data led us to propose a model of tungstate action (Figure 5) which could explain the general improvement in the exocrine and endocrine pancreatic function of the diabetic animals treated with sodium tungstate. To understand these effects, we should bear in mind that tungstate is able to inhibit phosphatase activity due to its chemical properties [21]. Our group and others have shown that, probably as a result of this property, tungstate treatment increases the phosphorylation of key proteins of different pathways [9,10] and is probably responsible for the effects of tungstate in diabetic animals. Thus, the combination of these two actions – modification of gene expression and enhancement of phosphorylation – may cooperate synergistically to increase beta cell mass, which, together with the improvement of hepatic metabolism [5], would lead to a decrease in hyperglycemia.

In relation to the recovery of exocrine function, we should mention the possible role of *Xbp1*. This gene plays a key role in the unfolded protein response [22], and *Xbp1 *knockout animals display abnormalities exclusively in secretory organs such as the exocrine pancreas, which lead to early postnatal lethality [23]. In our model, we found *Xbp1 *to be decreased in the DU animals and increased in the DT animals concomitantly to the recovery of the amylase expression, a finding that stresses the important role of this gene in the correct function of the exocrine machinery.

Several genes whose expression was modified by tungstate administration to diabetic animals may be responsible for the recovery of pancreatic function. Those genes are involved in different pathways which control a wide range of physiological functions, covering growth and differentiation to morphogenesis and angiogenesis. Therefore, it is probably the combination of tungstate effects upon all these pathways that leads to the recovery of pancreatic function. Since we performed microarrays using the total pancreas, we cannot identify the exact sites of these changes (exocrine, endocrine or ducts). Nevertheless, it seems that their modulation may contribute to the pancreatic regeneration observed in the diabetic treated animals. *Fgf13 *is one of the genes modified in the diabetic treated animals. Fgf13 binds to islet-brain 2, recruiting p38δ and increasing the activity of this kinase [24]. Interestingly, it has already been shown that tungstate increases the phosphorylation of p38 in both MIN6 beta cells [25] and islets of nSTZ-rats [9], which may lead to the improvement observed in the pancreatic beta cell population. Another modified gene was *Tspn8*, which may exert tumor-promoting activities by increasing cell motility and by inducing angiogenesis [26]. Therefore, bearing in mind that STZ damage provokes severe microcirculatory disturbances within pancreatic islets [27], we propose that an increase in the *Tspn8 *expression may improve pancreas revascularization and enhance islet function. Finally, other interesting genes are *Tgfb3, Usag-1 *and *Sel1h*. *Tgfb3 *plays a key role in complex processes including epithelial to mesenchymal transition [28], which is relatively important in pancreas regeneration [29,30]. *Usag-1 *modulates Wnt and Bone Morphogenic Protein signaling [31-33] in different ways. *Sel1h *has been implicated as a Notch inhibitor in exocrine [34] and endocrine replication [35] and modifies the expression of genes involved in cell-matrix interactions and in the cell cycle [36]. Although the exact role of these genes in pancreatic regeneration has not been investigated in detail, we hypothesize that the combined action of these multiple pathways leads to the regeneration of the pancreas observed in the DT rats.

Although many pathways are involved in tungstate action, the results clearly show that the MAPK pathway plays a key role. The array analysis showed normalization in *Rkip *expression in the DT animals. This protein binds to v-raf-1 murine leukemia viral oncogene homolog 1 (Raf-1), leading to the blockage of Mek and extracellular-signal-regulated kinase 1 (Erk) activation. It has been shown that, in the pancreas, Rkip localizes specifically in the islets and is involved in the regulation of beta cell growth. When this gene is up-regulated, as in the case of the DU animals, it acts as a brake on the MAPK signaling and beta cell proliferation [18]. The normalization of *Rkip *in the DT rats would permit the unblocking of the MAPK signaling and thus allowing the proliferative signals through this pathway. In the liver, it has been shown that tungstate enhances glycogen synthesis through MAPK activation [10]. Here we demonstrate that tungstate activates MAPK both directly and indirectly and is crucial for the tungstate-induced increase observed in beta cell replication. On the one hand, tungstate enhances MAPK phosphorylation; on the other, it normalizes *Rkip *expression which also leads to an increase in the MAPK pathway tone. The combination of these two mechanisms is the cause of the increased beta cell proliferation observed in our study.

## Conclusion

In summary, tungstate is able to increase beta cell mass and recovers the exocrine and partially endocrine function in STZ-rats. These improvements in pancreatic function require the combined action of several pathways, such as Wnt, TGF-b, Notch and MAPK. The last of these is clearly involved in the increase in beta cell mass. This study identifies several proteins that may play a key role in the improvement of pancreatic plasticity. Further studies will help us to identify their precise role in the pancreas.

## Methods

### Animals, induction of diabetes and tungstate treatment

Principles of laboratory animal care were followed (European and local government guidelines) and protocols were approved by the Animal Research Committee of the University of Barcelona. Diabetes induction and tungstate treatment have been described previously [8]. Diabetes was induced in adult male Wistar rats (225 g) obtained from Charles River (Wilmington, MA, USA) by an intraperitoneal STZ (Sigma, St Louis, MO, USA) injection (70 mg/Kg). At the beginning of the experiment, both diabetic and healthy animals were randomly divided into two groups. For 5 weeks, treated rats received *ad libitum *a solution of 2 mg/ml sodium tungstate (Carlo Erba, Rodano, Italy) in deionized water; whereas the untreated rats received deionized water alone (daily sodium tungstate intake: healthy 71 mg, diabetic 110 mg). Therefore, the study had 4 experimental groups (healthy untreated [HU], healthy treated [HT], diabetic untreated [DU] and diabetic treated [DT]). Glycemia was measured every 5 days. At the end of the experiment, blood was collected, and serum was obtained. For islet studies, islets were isolated by collagenase digestion, as previously described [9].

### Phloridzin administration

In an additional group of animals, diabetes was induced by STZ injection, as described in the previous section. Once the hyperglycemia was confirmed, these diabetic animals were subdivided into two groups, one group (control) which received the vehicle used to dissolve phloridzin; and the other group (treated) which received phloridzin as previously described [37]: phloridzin (Sigma) was dissolved (40%) in propylene glycol (Sigma) and injected intraperitoneally every 8 h during 1 week at a dose of 2 g/Kg body weight per day. Phloridzin treated rats also underwent a caloric restriction of 0.1 g chow diet/Kg body weight per day.

### Insulinemia and amylasemia

Insulinemia was measured by radioimmunoassay (CIS, Gif-sur-Yvette, France), and amylasemia was determined in an Advia Analyzer 2400 (Siemens Medical Solutions Diagnostics, Munich, Germany)

### Immunohistochemical studies and beta cell analysis

The whole pancreas was removed, fixed and embedded in paraffin. Serial sections of pancreas (5 μm) were obtained from three different levels. Beta cells were located using a modified avidin-biotin-peroxidase method [38]. The morphometric analysis was performed using OPTIMAS™ (Bioscan Incorporated, Edmonds, WA, USA) software. We also stained for pancreatic duodenal homeobox-1 (Pdx-1) (kindly provided by Dr. C. Wright), quantifying the percentage of Pdx-1-labeled cells within the islet cells (not less than 1000 cells were counted).

### Cell replication rates

Sequential-dual staining for proliferating cell nuclear antigen (PCNA) (Sigma) and insulin (Dako, Glostrup, Denmark) was performed. Replication rate was quantified and expressed as the percentage of PCNA-labeled cells among the total beta cells counted (not less than 3000 each). Immunohistochemical stainings have been validated in previous studies [39,40].

### Apoptotic rate

To identify apoptotic bodies, we used the propidium iodide technique [1]. Deparaffinized and rehydrated sections were incubated for 30 minutes in a solution of propidium iodide (4 μg/ml, Sigma) and RNAse A (100 μg/ml, Sigma). For the quantitative evaluation, positively labeled apoptotic endocrine cells were counted, and the number of apoptotic cells was expressed as the percentage of the total number of beta cells counted.

### RNA isolation

RNA was isolated from fresh total pancreas using the RNAgents Total RNA Isolation System (Promega, Madison, WI, USA). Islet RNA was isolated with the RNeasy Mini Kit (Qiagen, Venlo, The Netherlands). RNA integrity was analyzed using a Lab-on-a-chip in a 2100 Bioanalyzer (Agilent Technologies, Santa Clara, CA, USA).

### Microarrays

Total RNA from 3 rat pancreases of the same experimental group was pooled in equal proportions and hybridized to a Rat GeneChip RAE230-A (Affymetrix, Santa Clara, CA, USA). In total, 12 microarrays were hybridized using 3 different pools of each experimental group. The data were deposited in NCBIs Gene Expression Omnibus with the accession number GDS1883. Several quality controls were performed, as described previously [41] [see Additional file 5, 6, 7 and 8], and analyzed, as described in Additional file 9, using Affy [42,43], AffyPLM [44] and LIMMA [45] packages from Bioconductor [46] on R language [47,48] and dChip software [49].

Values for clustering were obtained after standardizing the gene values: the mean value of each gene was subtracted from the value of this gene in each array and was divided by the standard deviation of this gene in all the arrays. Clustering was performed with a distance metric of 1 – correlation, centroid linkage method and gene ordering by cluster tightness

### Real Time PCR

Total RNA was retrotranscripted with SuperscriptIII (Invitrogen, Carlsbad, CA, USA). Real Time PCR was carried out in a 7900 HT Real Time System (Applied Biosystems, Foster City, CA, USA) using SYBR Green fluorophor. The primers used are described in Additional file 10. A standard curve of each primer set was generated from serial dilutions of cDNA. The PCR products were verified by way of dissociation curve analysis using SDS software (Applied Biosystems). Expression levels obtained were normalized with a housekeeping gene (TATA box binding protein) and were scaled to the mean of the values of the same gene found in the healthy untreated animal.

### Western Blot

Western blotting was performed as described previously [9], using a specific antibody against phospho-p42-p44 *MAPK *(Thr202/Tyr204; Cell signaling, Danvers, MA, USA). Membranes were then stripped and incubated with anti-p42-p44 MAPK (Cell signaling). Intensity values were obtained with Image Gauge 4.0 software (Fujifilm, Valhalla, NY, USA). These values were divided by the value obtained at time 0, in order to normalize the data and permit comparisons among experiments performed on different days. Finally, data was log 2 transformed in order to obtain additive normally distributed noise and to stabilize variance [50].

### Cell proliferation

INS-1E cells (kindly provided by Dr P. Maechler) were maintained as described elsewhere [51]. For serum proliferation studies, two days after seeding, the medium was substituted by a medium containing RPMI1640 (Biosera, Ringmer, UK), BSA 0.1%, glucose 5 mmol/l and sodium tungstate at 100 μmol/l (Carlo Erba) or 10% of the serum from the animals. 24 hours later, hydroxyurea 12.5 mmol/l (Sigma) was added to the medium. 24 hours later, the medium was removed, and wells were cleaned with DPBS (Sigma). A medium with the initial composition supplemented with methyl-3H-Thymidine 1 μCi (initial specific activity 74 GBq/mmol) (GE Healthcare, Fairfield, CT, USA) was added to each well for 4 hours. Plates were then frozen and defrosted, cells were harvested with a Cell Harvester, and cpm was measured with a 1205 BetaPlate liquid scintillation counter (Perkin Elmer, Waltham, MA, USA).

### Phosphorylation studies

Two days after seeding INS-1E cells, in order to decrease basal phosphorylation levels, the medium was substituted by RPMI1640 without any supplement. Eight hours later, the medium was substituted by RPMI1640 complemented with 10% of the serum from animals of the different experimental groups or sodium tungstate (100 μmol/l). After 0, 5, 15, 30 and 60 minutes, the plates were rinsed with DPBS and frozen.

### Statistical analysis

Quantitative data are expressed as means ± SEM. The statistical significance was determined by Student's *t*-test (when only two groups were analyzed: glycaemia of the phloridizin treated animals, Real-time PCR values of the phloridizin treated animals and tungstate treated cells replication) and ANOVA (when more than two groups were analyzed: the rest), with a Tukey's Post-Hoc test used to find the groups responsible for the statistical significance.

## Authors' contributions

JA carried out the animal studies, the microarray and statistical analysis, the cellular treatments, the Real Time PCR and Western Blot experiments, and drafted the manuscript; HDZ, BN and JJG performed the pancreatic morphometrical studies; SP participated in the cellular treatments and Western Blot analysis; AS carried out the biostatistical analysis of the microarrays; AB helped in the animal treatments, AB and RG conceived the study and participated in its design and coordination; and JJG, AS, AB and RG helped to draft the manuscript. All authors read and approved the final manuscript.

## Supplementary Material

Additional file 1 **Differentially expressed genes in diabetic rats**. 282 genes differentially expressed due to diabetes and grouped according to their function. Click here for file

Additional file 2 **Differentially expressed genes in diabetic treated rats**. 88 genes differentially expressed in diabetic treated animals and grouped according to their function. Click here for file

Additional file 3 **Description of the specific pancreatic genes differentially expressed due to the treatment in diabetic animals**. 28 genes differentially expressed in diabetic treated animals alone with detailed information. These genes were selected from the list of differentially expressed genes in diabetic treated rats [see Additional file 2]. The selection criteria, in order to select those genes only significantly different in the treated diabetic animals, were that they were not present in the list of differentially expressed genes found in diabetic rats and that they presented a fold change higher than 1.25 or lower than -1.25 in the DT group, with respect to the HU group. The microarray expression values of these genes were represented using dChip. Its description includes its name, gene symbol and fold change compared to other experimental groups. The fold change between untreated healthy and untreated diabetic rats is reported in order to show that the differences in the gene's expression between the two groups are minimal. The identification of the genes differentially expressed was performed as described in Additional file 9. Click here for file

Additional file 4 **Genes selected for checking**. From the list of all the genes modified by tungstate in the diabetic animals we chose a selection of genes whose function would help us to explain the effects observed in the pancreas of the tungstate-treated diabetic animals. Here we describe why we selected these genes. Click here for file

Additional file 5 **Affymetrix quality controls of the microarrays**. Microarray quality controls according to the supplier. Click here for file

Additional file 6 **Statistical quality controls of the microarrays**. Microarray quality controls using different packages from Bioconductor. Click here for file

Additional file 7 **Raw and normalized data behaviour**. Figures described in the additional file 6. Histograms and boxplots of the raw data (A and B) and the background adjusted, normalized and summarized data by RMA (C and D). Click here for file

Additional file 8 **AffyPLM assessment quality control**. Figures described in the additional file 6. Boxplots of the normalized unscaled standard errors (NUSE boxplot, A) and boxplots of the distribution of the relative logarithmic expressions (RLE boxplot, B and C [magnificated]). Click here for file

Additional file 9 **Microarray analysis**. Explanation of the microarray analysis to obtain the differential expressed genes from each experimental group. Click here for file

Additional file 10 **Primers used in the Real Time PCR**. Description of the primers used in the Real Time PCR. Click here for file
