# Supplementary material for: Molecular mechanisms of tungstate-induced pancreatic plasticity: a transcriptomics approach
Source: BMC Genomics. 2009 Aug 28;10:406. doi: 10.1186/1471-2164-10-406 (PMC2741493; doi:10.1186/1471-2164-10-406)
Supplement: Additional file 1 — Differentially expressed genes in diabetic rats. 282 genes differentially expressed due to diabetes and grouped according to their function. [file 1471-2164-10-406-S1.pdf]

Differentially expressed genes in diabetic rats.

Genes up-regulated in STZ-diabetes			
**Probe set ID(affymetrix)**	**Gene name**	**Symbol**	**Relative change in diabetes**
**METABOLISM**			
**Lipid metabolism**			
1370310_at	3-hydroxy-3-methylglutaryl-Coenzyme A synthase 2	Hmgcs2	12.03
1367777_at	2,4-dienoyl CoA reductase 1, mitochondrial	Decr1	7.55
1386946_at	carnitine palmitoyltransferase 1a, liver	Cpt1a	4.83
1386885_at	enoyl coenzyme A hydratase 1, peroxisomal	Ech1	3.36
1367735_at	acetyl-Coenzyme A dehydrogenase, long-chain	Acadl	3.06
1370235_at	diazepam binding inhibitor	Dbi	2.56
1370237_at	L-3-hydroxyacyl-Coenzyme A dehydrogenase, short chain	Hadhsc	2.53
1367694_at	enoyl-Coenzyme A hydratase (trifunctional protein), beta subunit	Hadhb	2.16
1368283_at	enoyl-Coenzyme A, hydratase/3-hydroxyacyl Coenzyme A dehydrogenase	Ehhadh	2.08
1386901_at	similar to fatty acid translocase/CD36	RGD1562323	2.04
1370164_at	enoyl-Coenzyme A hydratase (trifunctional protein), alpha subunit	Hadha	1.96
1369150_at	pyruvate dehydrogenase kinase, isoenzyme 4	Pdk4	1.86
1386880_at	acetyl-Coenzyme A acyltransferase 2 (mitochondrial 3-oxoacyl-Coenzyme A thiolase)	Acaa2	1.73
1367662_at	hydroxyacyl-Coenzyme A dehydrogenase type II	Hadh2	1.67
1367638_at	malonyl-CoA decarboxylase	Mlycd	1.62
1373921_at	similar to enoyl Coenzyme A hydratase domain containing 3	LOC684538	1.57
1367702_at	acetyl-Coenzyme A dehydrogenase, medium chain	Acadm	1.56
1367767_at	3-hydroxy-3-methylglutaryl-Coenzyme A lyase	Hmgcl	1.52
1368717_at	fatty acid amide hydrolase	Faah	1.35
**Glucose metabolism**			
1368622_at	fructose-1,6-bisphosphatase 2	Fbp2	2.63
1368077_at	fructose-1,6-bisphosphatase 1	Fbp1	2.19

**Aminoacid metabolism**			
1371303_at	L-threonine dehydrogenase	Tdh	17.39
1370200_at	glutamate dehydrogenase 1	Glud1	1.81
**Other metabolisms**			
1373236_at	phosphopantothenoylcysteine decarboxylase	Ppcdc	5.87
1368037_at	carbonyl reductase 1	Cbr1	5.23
1370806_at	all-trans-13,14-dihydroretinol saturase	Retsat	2.36
1371031_at	methionine adenosyltransferase I, alpha	Mat1a	2.28
1370180_at	nudix (nucleoside diphosphate linked moiety X)-type motif 4	Nudt4	1.81
1389587_at	uridine monophosphate synthetase	Umps	1.45
**NUCLEAR PROTEINS**			
1371521_at	similar to Paired amphipathic helix protein Sin3b (Histone deacetylase complex subunit Sin3b)	LOC683381	2.90
1367847_at	nuclear protein 1	Nupr1	2.70
1371642_at	eukaryotic translation initiation factor 4A2	Eif4a2	1.96
1367798_at	S-adenosylhomocysteine hydrolase	Ahcy	1.94
1374731_at	Coilin	Coil	1.84
1398976_at	nuclear receptor co-repressor 1	Ncor1	1.64
1372242_at	DEAD/H (Asp-Glu-Ala-Asp/His) box polypeptide 3, X-linked	Ddx3x	1.55
1367485_at	transcription elongation factor A (SII) 1	Tcea1	1.54
1398878_at	similar to CPSF4 protein	MGC108785	1.51
1374954_at	histone deacetylase 11	Hdac11	1.50
1370258_at	basic leucine zipper and W2 domains 2	Bzw2	1.42
1373748_at	PDZ domain containing RING finger 3	Pdzrn3	1.39
1389738_at	uracil-DNA glycosylase	Ung	1.38
1388222_at	telomerase reverse transcriptase	Tert	1.38
1390573_a_at	nuclear factor of activated T-cells, cytoplasmic, calcineurin-dependent 4	Nfatc4	1.36
1374636_at	PHD finger protein 17	Phf17	1.35
1376023_at	similar to Retinoic acid receptor gamma-A (RAR-gamma-A)	LOC683586	1.30
**SIGNALLING**			
1387121_a_at	N-myc downstream regulated gene 2	Ndrg2	2.73
1371817_at	similar to myo-inositol 1-phosphate synthase A1	LOC290651	1.56

1367675_at	calcium and integrin binding 1 (calmyrin)	Cib1	1.52
1374550_at	similar to map kinase interacting kinase	MGC112775	1.52
1369312_a_at	casein kinase 1, alpha 1	Csnk1a1	1.49
1389420_at	signal-transducing adaptor protein-2	Stap2	1.47
1387342_at	guanine nucleotide binding protein (G protein), gamma 5 subunit	Gng5	1.43
1371994_at	Rho GTPase activating protein 1	Arhgap1	1.35
1398821_s_at	myosin light chain kinase 2, skeletal muscle	Mylk2	1.27
**PROTEIN MODIFICATION AND DEGRADATION**			
1388924_at	angiopoietin-like 4	Angptl4	3.17
1372536_at	chaperone, ABC1 activity of bc1 complex like (S. pombe)	Cabc1	1.78
1368446_at	serine protease inhibitor, Kazal type 1	Spink1	1.68
1387669_a_at	epoxide hydrolase 1, microsomal	Ephx1	1.63
1388470_at	F-box only protein 9	Fbxo9	1.53
1376035_at	tetratricopeptide repeat domain 7B (predicted)	Ttc7b_predicted	1.52
1398848_at	suppression of tumorigenicity 13	St13	1.48
**TRANSPORT / TRAFFICKING**			
1371782_at	nipsnap homolog 3A (C. elegans)	Nipsnap3a	1.81
1386937_at	ATPase, Na+/K+ transporting, beta 1 polypeptide	Atp1b1	1.70
1368418_a_at	ceruloplasmin	Cp	1.65
1387578_a_at	purinergic receptor P2X, ligand-gated ion channel, 2	P2rx2	1.55
1376874_a_at	adaptor-related protein complex AP-4, beta 1	Ap4b1	1.53
1389622_at	similar to citrin	RGD1565889	1.45
1367789_at	solute carrier family 27 (fatty acid transporter), member 1	Slc27a1	1.40
1374347_at	CDK5 regulatory subunit associated protein 1-like 1	Cdkal1	1.40
1390440_at	solute carrier family 9 (sodium/hydrogen exchanger), member 6	Slc9a6	1.38
1374692_at	Sorting nexin 14	Snx14	1.33
**OXIDATIVE STRESS**			
1367576_at	glutathione peroxidase 1	Gpx1	3.74
1371942_at	similar to Glutathione S-transferase, theta 3 (predicted)	RGD1562732_predicted	2.05
1389253_at	vanin 1	Vnn1	1.96
1398378_at	glutathione S-transferase kappa 1	Gstk1	1.94

**PHOSPHATASES**			
1392467_at	inositol (myo)-1(or 4)-monophosphatase 2	Impa2	3.45
1370193_at	protein tyrosine phosphatase 4a1	Ptp4a1	2.22
1369961_at	phosphatidic acid phosphatase 2a	Ppap2a	1.62
1373479_at	Protein phosphatase 3, catalytic subunit, alpha isoform	Ppp3ca	1.40
1388502_at	inositol polyphosphate-5-phosphatase B	Inpp5b	1.37
**CELL CYCLE / GROWTH**			
1371131_a_at	thioredoxin interacting protein	Txnip	5.96
1375420_at	tumor protein p53 inducible protein 11	Tp53i11	3.63
1367830_a_at	tumor protein p53	Tp53	1.71
1388953_at	guanine nucleotide binding protein-like 3 (nucleolar)	Gnl3	1.45
1372052_at	budding uninhibited by benzimidazoles 3 homolog (S. cerevisiae)	Bub3	1.45
1372133_at	related RAS viral (r-ras) oncogene homolog 2	Rras2	1.40
1376195_at	spindlin	Spin	1.36
**APOPTOSIS / DEFENSE RESPONSE**			
1398326_at	similar to Nur77 downstream protein 2	MGC105647	1.73
1398784_at	complement component 1, q subcomponent binding protein	C1qbp	1.58
1387805_at	BCL2/adenovirus E1B 19 kDa-interacting protein 3	Bnip3	1.40
**HORMONES**			
1367762_at	somatostatin	Sst	2.54
**EXOCRINE ENZYMES**			
1371092_at	preprotrypsinogen IV	LOC286960	8.06
**MEMBRANE**			
1388393_at	proteolipid protein 2	Plp2	1.99
1379429_at	Transmembrane emp24 protein transport domain containing 5	Tmed5	1.56
1375904_at	Transmembrane emp24 protein transport domain containing 5	Tmed5	1.56
1373058_at	transmembrane protein 30A	Tmem30a	1.52
1372369_at	transmembrane protein 109	Tmem109	1.41
1367746_a_at	flotillin 2	Flot2	1.41
**STRUCTURAL**			

1389533_at	fibulin 2	Fbln2	1.49
**MITOCHONDRIA**			
1372457_at	mitochondrial tumor suppressor 1	Mtus1	1.78
1389125_at	mitochondrial ribosomal protein L1 (predicted)	Mrpl1_predicted	1.45
**ELECTRON TRANSPORT**			
1388358_at	electron-transfer-flavoprotein, beta polypeptide	Etfb	1.82
1371253_at	electron transferring flavoprotein, alpha polypeptide	Etfa	1.71
1368336_at	ferredoxin 1	Fdx1	1.69
1371519_at	electron-transferring-flavoprotein dehydrogenase	Etfdh	1.64
1367705_at	glutaredoxin 1 (thioltransferase)	Glrx1	1.39
1370005_at	cytochrome b5 type B	Cyb5b	1.39
**OTHERS**			
1373898_at	similar to PABP-dependent poly(A) nuclease 3	LOC360760	1.64
1372688_at	exosome component 7	Exosc7	1.33
**ESTS**			
1390249_at	similar to DKFZP434H132 protein	RGD1305464	7.78
1372372_at	similar to Ab2-225	RGD1306952	2.38
1399001_at	hypothetical LOC294030	RGD1305481	2.26
1373778_at	Transcribed locus		2.07
1374816_at	similar to hypothetical protein FLJ30973	LOC363091	1.92
1377460_at	EST		1.89
1371445_at	leucine rich repeat containing 59	Lrrc59	1.70
1371976_at	hypothetical protein LOC682248	LOC682248	1.68
1371854_at	EST		1.65
1367494_at	similar to CGI-35 protein (predicted)	RGD1310899_predicted	1.56
1372027_at	Transcribed locus		1.54
1372843_at	LOC363020 (predicted)	RGD1309410_predicted	1.52
1374194_at	EST		1.50
1375934_at	similar to RIKEN cDNA D330045A20 (predicted)	RGD1566282_predicted	1.50
1371856_at	CDNA clone MGC:93828 IMAGE:7110880		1.50
1388739_at	similar to chromosome 14 open reading frame 130	RGD1359144	1.48

1388887_at	Transcribed locus		1.47
1371631_at	similar to 2010003J03Rik protein	RGD1304686	1.45
1376034_at	similar to mKIAA0738 protein (predicted)	RGD1565474_predicted	1.44
1373497_at	Transcribed locus		1.43
1373033_at	similar to chromosome 6 open reading frame 83; similar to RIKEN cDNA 5630401J11	MGC105508	1.43
1373334_at	similar to RIKEN cDNA 0610039G24 gene (predicted)	RGD1310828_predicted	1.43
1388579_at	similar to alternative testis transcripts open reading frame A CG4241-PA, isoform A	LOC684597	1.42
1385866_at	Transcribed locus		1.42
1373922_at	Transcribed locus		1.41
1390288_at	EST		1.36
1374090_at	similar to DKFZP547E1010 protein	LOC361990	1.35

Genes down-regulated in STZ-diabetes

Probe set ID (affymetrix)	Gene name	Symbol	Relative change in diabetes
**METABOLISM**			
**Lipid metabolism**			
1367887_at	lecithin cholesterol acyltransferase	Lcat	-4.09
1368453_at	fatty acid desaturase 2	Fads2	-1.81
1388148_a_at	low density lipoprotein receptor-related protein associated protein 1	Lrpap1	-1.76
1388190_at	apolipoprotein B	Apob	-1.72
1373053_at	Similar to Acyl-CoA-binding protein (ACBP)		-1.71
1368016_at	peroxisomal trans-2-enoyl-CoA reductase	Pecr	-1.46
1372443_at	low density lipoprotein receptor-related protein 11	Lrp11	-1.40
**Aminoacid metabolism**			
1387034_at	phenylalanine hydroxylase	Pah	-1.83

1372741_at	saccharopine dehydrogenase (putative)	Sccpdh	-1.34
**Other metabolisms**			
1387022_at	aldehyde dehydrogenase family 1, member A1	Aldh1a1	-2.44
1367999_at	aldehyde dehydrogenase 2	Aldh2	-1.53
1373222_at	hexosaminidase A	Hexa	-1.53
1387339_at	selenoprotein P, plasma, 1	Sepp1	-1.45
1369863_at	alcohol dehydrogenase 4 (class II), pi polypeptide	Adh4	-1.35
1388330_at	vitamin K epoxide reductase complex, subunit 1	Vkorc1	-1.35
**NUCLEAR PROTEINS**			
1371959_at	histone cluster 2, H2aa	Hist2h2aa	-3.72
1373860_at	SRY-box containing gene 4	Sox4	-2.03
1390021_at	histone cluster 1, H2bh	Hist1h2bh	-1.91
1368376_at	nuclear receptor subfamily 0, group B, member 2	Nr0b2	-1.74
1374600_at	histone cluster 1, H4b	Hist1h4b	-1.66
1383065_at	nicolin 1	Nicn1	-1.42
1387278_at	peroxisome proliferator activated receptor alpha	Ppara	-1.40
1370386_at	RuvB-like protein 1	Ruvbl1	-1.39
1369085_s_at	small nuclear ribonucleoprotein N	Snrpn	-1.38
1369728_at	similar to germinal histone H4 gene	LOC680097	-1.30
**SIGNALLING**			
1398296_at	membrane interacting protein of RGS16	Mir16	-2.97
1370150_a_at	thyroid hormone responsive protein	Thrsp	-2.77
1387671_at	secretin receptor	Sctr	-2.10
1368884_at	ectonucleoside triphosphate diphosphohydrolase 1	Entpd1	-1.70
1370842_at	branched chain ketoacid dehydrogenase kinase	Bckdk	-1.70
1368523_at	Ca2+-dependent secretion activator	Cadps	-1.68
1367905_at	ectonucleotide pyrophosphatase/phosphodiesterase 3	Enpp3	-1.64
1370948_a_at	myristoylated alanine rich protein kinase C substrate	LOC294446	-1.60
1369855_at	histamine receptor H 1	Hrh1	-1.58
1398298_at	5-hydroxytryptamine (serotonin) receptor 1D	Htr1d	-1.54
1371158_at	Eph receptor A8	Epha8	-1.49

1375566_at	angiotensin II, type I receptor-associated protein	Agtrap	-1.46
1373365_at	cytidylate kinase	Cmpk	-1.46
1369230_at	gamma-aminobutyric acid (GABA-C) receptor, subunit rho 2	Gabrr2	-1.43
**PROTEIN MODIFICATION AND DEGRADATION**			
1369286_at	protein C	Proc	-2.46
1388699_at	mannosidase 2, alpha B1	Man2b1	-2.39
1371615_at	diacylglycerol O-acyltransferase homolog 2 (mouse)	Dgat2	-2.39
1372421_at	aspartylglucosaminidase	Aga	-2.05
1387131_at	serine (or cysteine) peptidase inhibitor, clade I, member 1	Serpini1	-2.03
1376249_at	fucosidase, alpha-L- 2, plasma	Fuca2	-1.96
1367835_at	proprotein convertase subtilisin/kexin type 1 inhibitor	Pcsk1n	-1.85
1392798_at	protein disulfide isomerase associated 2	Pdia2	-1.78
1387155_at	proprotein convertase subtilisin/kexin type 2	Pcsk2	-1.74
1372706_at	hexosaminidase B	Hexb	-1.72
1386938_at	alanyl (membrane) aminopeptidase	Anpep	-1.71
1374790_at	N-acetylglucosamine-1-phosphotransferase, gamma subunit	Gnptg	-1.68
1371358_at	glycoprotein, synaptic 2	Gpsn2	-1.67
1372504_at	similar to dolichyl-phosphate mannosyltransferase polypeptide 3 (Dolichol-phosphate mannose synthase subunit 3)	LOC684429	-1.57
1388643_at	fucosyltransferase 8 (alpha (1,6) fucosyltransferase)	Fut8	-1.39
1391272_at	similar to WAP four-disulfide core 6-like 1	LOC684696	-1.36
1389833_at	Sulfatase modifying factor 1	Sumf1	-1.34
1368281_at	dipeptidase 1 (renal)	Dpep1	-1.34
**TRANSPORT / TRAFFICKING**			
1368044_at	secretogranin 2	Scg2	-6.95
1387228_at	solute carrier family 2 (facilitated glucose transporter), member 2	Slc2a2	-3.68
1368316_at	aquaporin 8	Aqp8	-2.90
1369999_a_at	neuronatin	Nnat	-2.13
1369769_at	potassium voltage-gated channel, Isk-related subfamily, member 1	Kcne1	-1.84
1374427_at	Synaptotagmin XIII	Syt13	-1.57
1367877_at	solute carrier family 11 (proton-coupled divalent metal ion transporters), member 2	Slc11a2	-1.49
1376413_at	Amyloid beta (A4) precursor protein-binding, family A, member 1	Apba1	-1.43

1371889_at	solute carrier family 22 (organic cation transporter), member 17	Slc22a17	-1.37
1368343_at	potassium voltage-gated channel, subfamily H (eag-related), member 2	Kcnh2	-1.36
**OXIDATIVE STRESS**			
1367917_at	cytochrome P450, family 2, subfamily d, polypeptide 26	Cyp2d26	-2.49
1368023_at	quiescin Q6	Qscn6	-2.18
1388122_at	glutathione-S-transferase, pi 1 /// glutathione S-transferase, pi 2	Gstp1 /// Gstp2	-1.96
1386940_at	tissue inhibitor of metalloproteinase 2	Timp2	-1.90
1368514_at	monoamine oxidase B	Maob	-1.72
1373043_at	stromal cell-derived factor 2-like 1 (predicted)	Sdf2l1	-1.70
1375357_at	dystonia 1	Dyt1	-1.51
1368265_at	cytochrome P450 monooxygenase CYP2T1	Cyp2t1	-1.49
1371249_at	X-box binding protein 1	Xbp1	-1.48
1387305_s_at	cytochrome P450, family 11, subfamily B, polypeptide 2 /// cytochrome P450, subfamily 11B, polypeptide 1	Cyp11b1 /// Cyp11b2	-1.34
**PHOSPHATASES**			
1386968_at	protein phosphatase 1, regulatory (inhibitor) subunit 1A	Ppp1r1a	-1.86
1370182_at	protein tyrosine phosphatase, receptor type, N polypeptide 2	Ptprn2	-1.40
1388805_at	Protein phosphatase 2 (formerly 2A), catalytic subunit, alpha isoform	Ppp2ca	-1.40
1388048_a_at	inositol polyphosphate-4-phosphatase, type II	Inpp4b	-1.29
**CELL CYCLE / GROWTH**			
1376073_at	Sel1 (suppressor of lin-12) 1 homolog	Sel1h	-2.06
1374357_at	CDC91 cell division cycle 91-like 1	Cdc91l1	-1.37
**APOPTOSIS / DEFENSE RESPONSE**			
1370027_a_at	alpha-1-inhibitor III	LOC297568	-9.05
1388229_a_at	Murinoglobulin 1 homolog (mouse)	Mug1	-4.45
1369953_a_at	CD24 antigen	Cd24	-2.83
1389762_at	similar to Fc fragment of IgG binding protein	RGD1311906	-2.37
1370243_a_at	prothymosin alpha	Ptma	-1.69
1369879_a_at	testis enhanced gene transcript	Tegt	-1.63
**HORMONES**			
1370077_at	insulin 2	Ins2	-25.73

1387815_at	insulin 1	Ins1	-7.72
1387660_at	islet amyloid polypeptide	Iapp	-4.39
1367904_at	regulated endocrine-specific protein 18	Resp18	-2.17
1368312_at	oxytocin	Oxt	-1.52
**EXOCRINE ENZYMES**			
1370359_at	amylase 1, salivary	Amy1	-68.66
1369503_at	amylase 2, pancreatic	Amy2	-29.37
1390326_at	angiogenin, ribonuclease A family, member 1	Ang1	-2.89
1386921_at	carboxypeptidase E	Cpe	-3.78
1387503_at	carboxypeptidase N, polypeptide 1, 50kD	Cpn1	-1.41
**MEMBRANE**			
1398325_at	tetraspanin 3	Tspan3	-2.25
1387135_at	a disintegrin and metallopeptidase domain 15 (metargidin)	Adam15	-1.58
1372780_at	transmembrane protein 53	Tmem53	-1.37
**MITOCHONDRIA**			
1375788_at	Ribosomal protein L7	Rpl7	-2.37
1398946_at	similar to mitochondrial ribosomal protein S16	LOC683012	-1.44
**STRUCTURAL**			
1368113_at	trefoil factor 2 (spasmolytic protein 1)	Tff2	-40.39
1388793_at	phosphatidylinositol glycan, class Q	Pigq	-1.54
1374399_at	EH-domain containing 4	Ehd4	-1.50
1387844_at	LIM and SH3 protein 1	Lasp1	-1.42
1368367_at	CUB and zona pellucida-like domains 1	Cuzd1	-1.42
**OTHERS**			
1388723_at	brain and reproductive organ-expressed protein	Bre	-1.55
1378983_at	Similar to translin-associated factor X (Tsnax) interacting protein 1	RGD1565341	-1.49
1373438_at	ubiquitin-conjugating enzyme E2O	Ube2o	-1.44
1374287_at	paladin	Pald	-1.40
**ESTS**			
1388985_at	Transcribed locus, moderately similar to NP_001020173.1 protein LOC310926		-3.97

1381975_at	Similar to kIAA0367	RGD1311350	-2.27
1373908_at	EST		-1.93
1373114_at	Transcribed locus		-1.81
1385507_at	Transcribed locus		-1.80
1383165_at	EST		-1.79
1390153_at	Transcribed locus		-1.74
1388447_at	similar to limb-bud and heart	LOC683626	-1.74
1383162_at	EST		-1.73
1376861_at	similar to RIKEN cDNA 1810018L05	LOC317312	-1.68
1374124_at	similar to RIKEN cDNA 2610003J06	RGD1307381	-1.60
1383161_a_at	EST		-1.59
1389905_at	EST		-1.56
1368226_at	Nucleoside 2-deoxyribosyltransferase domain containing protein RGD620382	RGD620382	-1.55
1390733_at	WW domain-containing oxidoreductase (predicted)	Wwox_predicted	-1.55
1371399_at	hypothetical protein LOC681123	LOC681123	-1.53
1377753_at	Par-6 partitioning defective 6 homolog gamma (C. elegans) (predicted)	Pard6g_predicted	-1.50
1371663_at	similar to expressed sequence AW556797 (predicted)	RGD1305138_predicted	-1.48
1389395_at	Transcribed locus		-1.44
1377199_at	EST		-1.41
1377773_at	Transcribed locus, weakly similar to NP_766020.1 cDNA C030002O17 [Mus musculus]		-1.41
1375708_at	EST		-1.40
1392784_at	EST		-1.39
1371298_at	CDNA clone IMAGE:7460165		-1.39
1371881_at	zinc finger, HIT domain containing 2	Znhit2	-1.39
1370947_at	hypothetical protein RDA279	Rda279	-1.38
1371597_at	ring finger protein 187 (predicted)	Rnf187_predicted	-1.36
1376391_at	Transcribed locus		-1.34
1371468_at	similar to RAB11 family interacting protein 5 (class I) isoform 1	LOC312502	-1.32
1388402_at	similar to 2410001H17Rik protein (predicted)	RGD1305824_predicted	-1.31
1375648_at	similar to hypothetical protein	RGD1307966	-1.30
1375116_at	Hypothetical protein LOC690771	LOC690771	-1.29
