# Supplementary material for: Molecular mechanisms of tungstate-induced pancreatic plasticity: a transcriptomics approach
Source: BMC Genomics. 2009 Aug 28;10:406. doi: 10.1186/1471-2164-10-406 (PMC2741493; doi:10.1186/1471-2164-10-406)
Supplement: Additional file 2 — Differentially expressed genes in diabetic treated rats. 88 genes differentially expressed in diabetic treated animals and grouped according to their function. [file 1471-2164-10-406-S2.pdf]

Differentially expressed genes in diabetic treated rats.

Genes up-regulated in treated diabetic rats				
Probe set ID(affymetrix)	Gene name	Symbol	Relative change	
			DT vs DU	DT vs H
**METABOLISM**				
**Aminoacid metabolism**				
1389785_at	aspartoacylase (aminoacylase) 3	Acy3	1.91	1.81
1387034_at	phenylalanine hydroxylase	Pah	1.90	1.04
1368344_at	glutamic acid decarboxylase 1	Gad1	1.80	1.32
1377375_at	aminoadipate-semialdehyde synthase	Aass	1.52	-1.01
**Other metabolisms**				
1371774_at	spermidine/spermine N1-acetyl transferase	Sat	2.88	2.86
**NUCLEAR PROTEINS**				
1371959_at	histone cluster 2, H2aa	Hist2h2aa	2.41	-1.55
1368376_at	nuclear receptor subfamily 0, group B, member 2	Nr0b2	1.81	1.04
1374600_at	histone cluster 1, H4b	Hist1h4b	1.55	-1.07
**SIGNALLING**				
1379281_at	uterine sensitization-associated protein-1	Usag-1	2.17	1.45
1367905_at	ectonucleotide pyrophosphatase/phosphodiesterase 3	Enpp3	1.73	1.06
1398298_at	5-hydroxytryptamine (serotonin) receptor 1D	Htr1d	1.61	1.05
1368705_at	endothelial differentiation, sphingolipid G-protein-coupled receptor, 8	Edg8	1.50	1.28
**PROTEIN MODIFICATION AND DEGRADATION**				
1373381_at	hect domain and RLD 4	Herc4	6.45	4.02
1386938_at	alanyl (membrane) aminopeptidase	Anpep	2.96	1.73
1388680_at	C1GALT1-specific chaperone 1	C1galt1c1	1.74	1.33
1368281_at	dipeptidase 1 (renal)	Dpep1	1.59	1.19
1368131_at	calpain 6	Capn6	1.56	1.26
1369286_at	protein C	Proc	1.42	-1.73
1368430_at	legumain	Lgmn	1.40	1.53

**TRANSPORT / TRAFFICKING**				
1368316_at	aquaporin 8	Aqp8	2.27	-1.28
1376709_at	solute carrier family 39 (metal ion transporter), member 8	Slc39a8	1.80	1.40
1369769_at	potassium voltage-gated channel, Isk-related subfamily, member 1	Kcne1	1.66	-1.11
1372710_at	blocked early in transport 1 homolog (S. cerevisiae)	Bet1	1.41	1.35
1388163_at	solute carrier family 25 (mitochondrial carrier; adenine nucleotide translocator), member 5	Slc25a5	1.40	1.39
**OXIDATIVE STRESS**				
1367917_at	cytochrome P450, family 2, subfamily d, polypeptide 26	Cyp2d26	2.63	1.06
1368023_at	quiescin Q6	Qscn6	1.94	-1.12
1388122_at	glutathione-S-transferase, pi 1 /// glutathione S-transferase, pi 2	Gstp1 /// Gstp2	1.90	-1.03
1371249_at	X-box binding protein 1	Xbp1	1.75	1.18
1398857_at	surfeit 1	Surf1	1.49	1.04
**CELL CYCLE / GROWTH**				
1367859_at	transforming growth factor, beta 3	Tgfb3	2.37	1.82
1369953_a_at	CD24 antigen	Cd24	2.37	-1.19
1376073_at	Sel1 (suppressor of lin-12) 1 homolog	Sel1h	2.24	1.09
1368114_at	fibroblast growth factor 13	Fgf13	2.23	1.49
1373043_at	stromal cell-derived factor 2-like 1	Sdf2l1	1.98	1.17
1371360_at	N-myc downstream regulated gene 1	Ndrg1	1.52	1.29
**APOPTOSIS / DEFENSE RESPONSE**				
1370243_a_at	prothymosin alpha	Ptma	1.39	-1.21
**MEMBRANE**				
1368052_at	tetraspanin 8	Tspan8	2.78	2.41
1388545_at	SPARC-related modular calcium binding protein 1	Smoc1	2.02	2.24
**EXOCRINE ENZYMES**				
1369503_at	amylase 2, pancreatic	Amy2	26.21	-1.12
1370359_at	amylase 1, salivary	Amy1	15.67	-4.38
1390326_at	angiogenin, ribonuclease A family, member 1	Ang1	2.39	-1.21
1368943_at	ribonuclease, RNase A family 4	Rnase4	1.83	1.38
1370880_at	ribonuclease/angiogenin inhibitor 1	Rnh1	1.32	1.14

**STRUCTURAL**				
1368113_at	trefoil factor 2 (spasmolytic protein 1)	Tff2	30.67	-1.32
1368367_at	CUB and zona pellucida-like domains 1	Cuzd1	1.70	1.19
1368099_at	CLIP associating protein 2	Clasp2	1.40	1.43
**OTHERS**				
1389654_at	Plastin 1	Pls1_predicted	1.58	1.29
**ESTS**				
1375052_at	similar to nuclear localized factor 2	LOC501015	3.97	3.54
1389762_at	similar to Fc fragment of IgG binding protein	RGD1311906	2.56	1.08
1383606_at	membrane targeting (tandem) C2 domain containing 1	Mtac2d1	1.56	2.05
1374349_at	CTD (carboxy-terminal domain, RNA polymerase II, polypeptide A) small phosphatase-like	Ctdspl_predicted	1.54	1.32
1388900_at	RGD1566118 (predicted)	RGD1566118_predicted	1.54	1.29
1389732_at	Similar to CG4025-PA	LOC679937	1.49	1.51
1373705_at	similar to 39S ribosomal protein L28, mitochondrial precursor (L28mt) (MRP-L28)	LOC497876	1.40	1.17
1372642_at	Transcribed locus		1.28	1.44

Genes down-regulated in treated diabetic rats

Probe set ID(affymetrix)	Gene name	Symbol	Relative change	
			DT vs DU	DT vs HU
**METABOLISM**				
**Lipid metabolism**				
1370310_at	3-hydroxy-3-methylglutaryl-Coenzyme A synthase 2	Hmgcs2	-11.81	1.02
1367777_at	2,4-dienoyl CoA reductase 1, mitochondrial	Decr1	-4.96	1.52
1386885_at	enoyl coenzyme A hydratase 1, peroxisomal	Ech1	-3.35	1.00
1370237_at	L-3-hydroxyacyl-Coenzyme A dehydrogenase, short chain	Hadhsc	-2.72	-1.08
1367735_at	acetyl-Coenzyme A dehydrogenase, long-chain	Acadl	-2.44	1.25

1386901_at	similar to fatty acid translocase/CD36	RGD1562323	-2.14	-1.05
1367694_at	hydroxyacyl-Coenzyme A dehydrogenase	Hadhb	-1.96	1.10
1368283_at	enoyl-Coenzyme A, hydratase/3-hydroxyacyl Coenzyme A dehydrogenase	Ehhadh	-1.85	1.13
**Glucose metabolism**				
1368622_at	fructose-1,6-bisphosphatase 2	Fbp2	-2.75	-1.04
1368077_at	fructose-1,6-bisphosphatase 1	Fbp1	-2.07	1.06
**Aminoacid metabolism**				
1371303_at	L-threonine dehydrogenase	Tdh	-31.68	-1.82
**Other metabolisms**				
1373236_at	phosphopantothenoylcysteine decarboxylase	Ppcdc	-6.32	-1.08
1368037_at	carbonyl reductase 1	Cbr1	-4.52	1.16
1370806_at	all-trans-13,14-dihydroretinol saturase	Retsat	-2.98	-1.27
**NUCLEAR PROTEINS**				
1371521_at	similar to Paired amphipathic helix protein Sin3b (Histone deacetylase complex subunit Sin3b)	LOC683381	-2.25	1.29
**SIGNALLING**				
1389420_at	signal-transducing adaptor protein-2	Stap2	-1.39	1.06
**PROTEIN MODIFICATION AND DEGRADATION**				
1388924_at	angiopoietin-like 4	Angptl4	-3.37	-1.06
1372536_at	chaperone, ABC1 activity of bc1 complex like	Cabc1	-2.06	-1.15
**TRANSPORT / TRAFFICKING**				
1368418_a_at	ceruloplasmin	Cp	-1.42	1.17
**OXIDATIVE STRESS**				
1389253_at	vanin 1	Vnn1	-2.30	-1.18
1371942_at	similar to Glutathione S-transferase, theta 3	RGD1562732	-2.15	-1.05
**PHOSPHATASES**				
1392467_at	inositol (myo)-1(or 4)-monophosphatase 2	Impa2	-3.21	1.07
**CELL CYCLE / GROWTH**				
1371131_a_at	thioredoxin interacting protein	Txnip	-4.56	1.31
1386891_at	Raf kinase inhibitory protein	Rkip	-1.59	-1.29

**OTHERS**				
1368000_at	complement component 3	C3	-1.32	1.00
**ESTS**				
1390249_at	similar to DKFZP434H132 protein	RGD1305464	-5.57	1.40
1372372_at	similar to Ab2-225	RGD1306952	-3.40	-1.43
1377460_at	EST		-1.88	1.01
1371922_at	Transcribed locus		-1.54	-1.25
1373898_at	similar to PABP-dependent poly(A) nuclease 3	LOC360760	-1.42	1.16
1373117_at	ubiquitin-like, containing PHD and RING finger domains 2 (predicted)	Uhrf2_predicted	-1.36	-1.06
1377208_at	Transcribed locus		-1.35	-1.07
1376931_at	similar to Hepatocellular carcinoma-associated antigen 58 homolog (predicted)	RGD1305020_predicted	-1.32	-1.07
