# Supplementary figures and images for: Molecular mechanisms of tungstate-induced pancreatic plasticity: a transcriptomics approach
Source: BMC Genomics. 2009 Aug 28;10:406. doi: 10.1186/1471-2164-10-406 (PMC2741493; doi:10.1186/1471-2164-10-406)

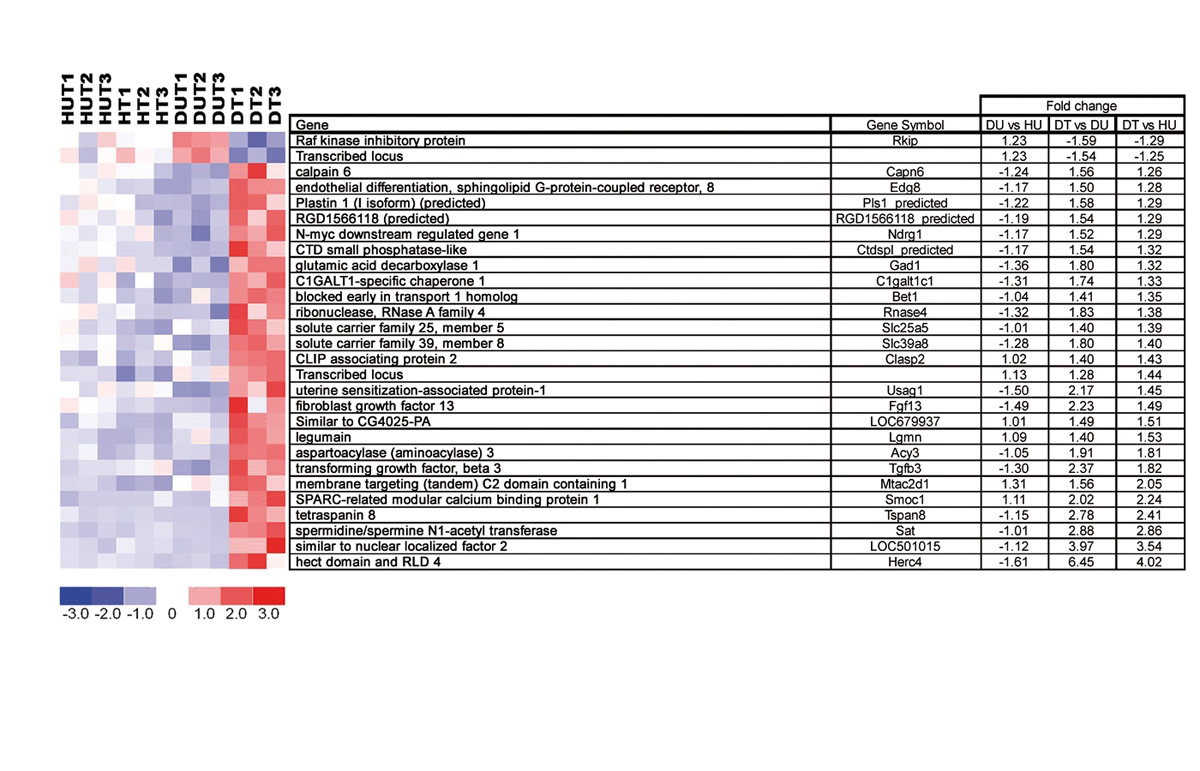

Supplement: Additional file 3 — Description of the specific pancreatic genes differentially expressed due to the treatment in diabetic animals. 28 genes differentially expressed in diabetic treated animals alone with detailed information. These genes were selected from the list of differentially expressed genes in diabetic treated rats [see Additional file 2]. The selection criteria, in order to select those genes only significantly different in the treated diabetic animals, were that they were not present in the list of differentially expressed genes found in diabetic rats and that they presented a fold change higher than 1.25 or lower than -1.25 in the DT group, with respect to the HU group. The microarray expression values of these genes were represented using dChip. Its description includes its name, gene symbol and fold change compared to other experimental groups. The fold change between untreated healthy and untreated diabetic rats is reported in order to show that the differences in the gene's expression between the two groups are minimal. The identification of the genes differentially expressed was performed as described in Additional file 9. [file 1471-2164-10-406-S3.jpeg]

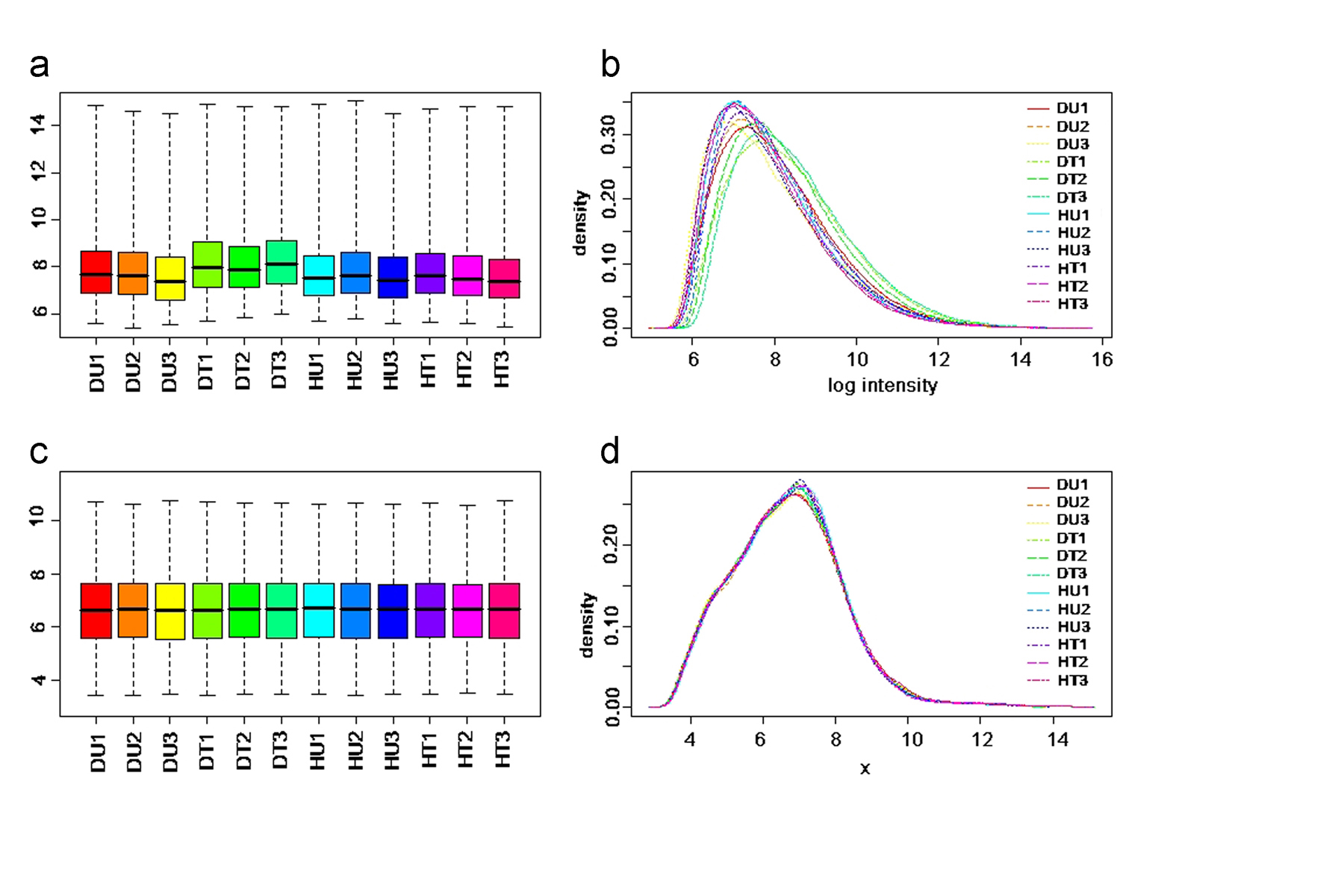

Supplement: Additional file 7 — Raw and normalized data behaviour. Figures described in the additional file 6. Histograms and boxplots of the raw data (A and B) and the background adjusted, normalized and summarized data by RMA (C and D). [file 1471-2164-10-406-S7.jpeg]

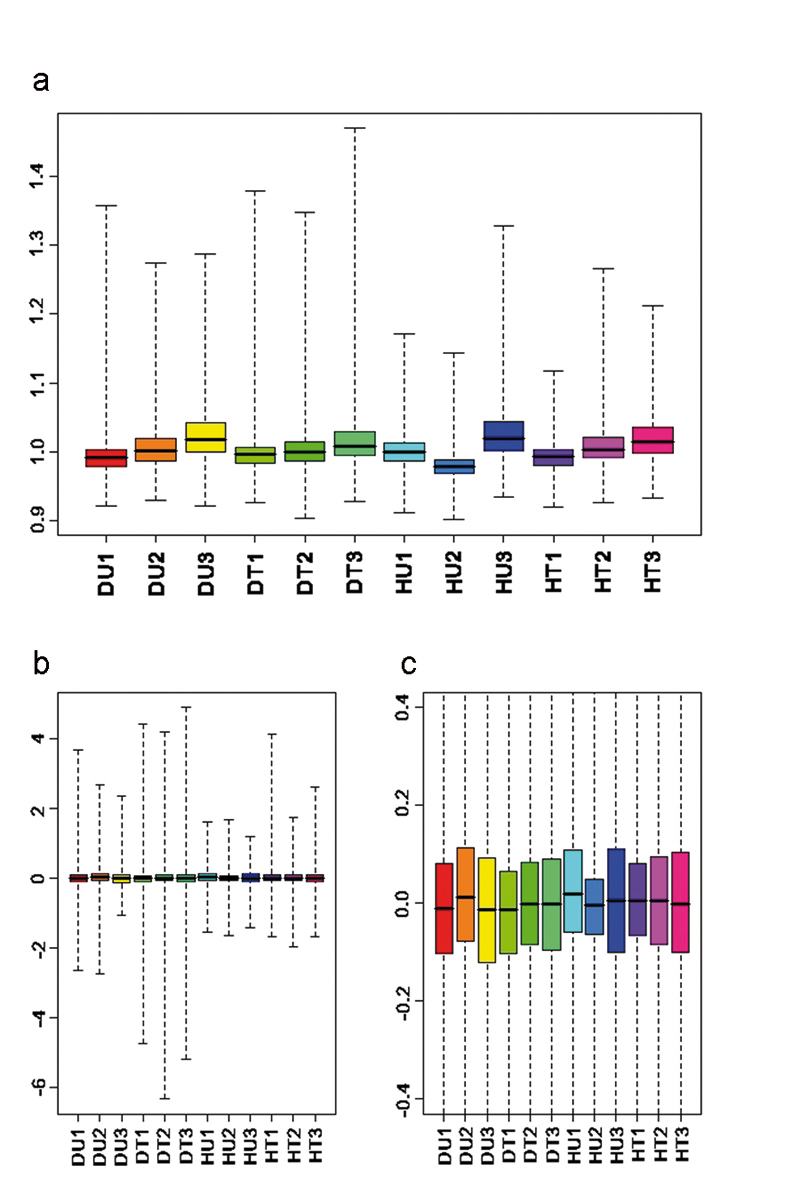

Supplement: Additional file 8 — AffyPLM assessment quality control. Figures described in the additional file 6. Boxplots of the normalized unscaled standard errors (NUSE boxplot, A) and boxplots of the distribution of the relative logarithmic expressions (RLE boxplot, B and C [magnificated]). [file 1471-2164-10-406-S8.jpeg]
