# Supplementary material for: Molecular mechanisms of tungstate-induced pancreatic plasticity: a transcriptomics approach
Source: BMC Genomics. 2009 Aug 28;10:406. doi: 10.1186/1471-2164-10-406 (PMC2741493; doi:10.1186/1471-2164-10-406)
Supplement: Additional file 4 — Genes selected for checking. From the list of all the genes modified by tungstate in the diabetic animals we chose a selection of genes whose function would help us to explain the effects observed in the pancreas of the tungstate-treated diabetic animals. Here we describe why we selected these genes. [file 1471-2164-10-406-S4.pdf]

Genes selected for checking.

	Why these genes are selected?	Pathway	References
**Rkip**	• In the pancreas, localizes specifically in the islets<br>• Controls beta cell proliferation<br>• RKIP binds to Raf-1, leading to the blockage of Mek and Erk activation<br>• Tungstate activates the classical MAPK pathway (Ras->Raf->Mek->Erk) in hepatocytes	MAPK p42/44	• Surgery 136:708-715<br>• J Biol Chem 278:42785-42794
**Fgf13**	• Fgf13 binds to islet-brain 2, recruiting p38δ, and increasing the activity of this kinase<br>• Tungstate increases the phosphorylation of p38 in the islets of nSTZ-rats	MAPK p38	• J Biol Chem 277:49111-49119<br>• Diabetologia 47:470- 477
**Tspn8**	• Tetraspanin 8 may exert tumor-promoting activities through an increase in cell motility and by inducing angiogenesis<br>• STZ damage provokes a reduction in islet capillary area and severe microcirculatory disturbances within pancreatic islets		• Am J Physiol 273:E376-E382<br>• Cancer Res 66:7083-7094
**Usag-1**	• Inhibits or increases Wnt signaling depending on the context and inhibits BMP signaling<br>• Controls the cell fate of cells in the kidney, tooth and hair<br>• Wnt and BMP signaling have been demonstrated to be essential for the development and regeneration of the pancreas	Wnt<br>BMP	• Development. 130:4295-305<br>• Cytokine Growth Factor Rev. 16:309-17<br>• Kidney Int. 73:181-91<br>• Science. 309:2067-70<br>• Proc Natl Acad Sci U S A. 102:14653-8<br>• Proc Natl Acad Sci U S A. 104:6247-52<br>• BMC Dev Biol. 7:4<br>• Gastroenterology. 134:544-55<br>• J Cell Sci. 115:753-60
**Tgfb3**	• Controls epithelial mesenchymal transformation, mesenchymal proliferation and angiogenesis during mouse palate development<br>• Regulates neuron differentiation and blood-testis barrier dynamics through the Sertoli junctions<br>• Other members of the TGF family have biological activities that also control adhesion, proliferation, and differentiation, which are important processes for pancreas regeneration	Tgf-beta	• Plast Reconstr Surg 108:938-948<br>• J Cell Biol. 163:1291-301<br>• Birth Defects Res A Clin Mol Teratol. 73:956-65<br>• Stem Cells. 24:2120-9<br>• Biol Reprod. 68:1597-612<br>• Annu Rev Biochem. 67:753-91<br>• Science 306:2261-2264<br>• Mol. Endocrinol 21:1467-477
**Xbp1**	• Key role in the unfolded protein response<br>• Xbp1 knockout animals display abnormalities exclusively in secretory organs such as exocrine pancreas which lead to early postnatal lethality	UPR (Unfolding protein response)	• Mol Cell Biol 23:7448-7459<br>• EMBO J 24:4368-80
**Sel1h**	• Present in the acini and the islet in the pancreas<br>• Implicated in beta-cell growth<br>• Inhibits Notch pathway<br>• Can modulate the TGFbeta pathway<br>• Both pathways, TGFbeta and Notch pathway, can control the cell fate and are essential for the pancreas development	UPR (Unfolding protein response)<br>Notch<br>Tgf-beta	• Oncogene 22:6359-68<br>• DNA Cell Biol. 23:510-518<br>• FEBS Lett. 581:5355-60<br>• Res Commun Mol Pathol Pharmacol. 102:265-72<br>• Am J Pathol 169:1206-1214
