# Supplementary material for: Molecular mechanisms of tungstate-induced pancreatic plasticity: a transcriptomics approach
Source: BMC Genomics. 2009 Aug 28;10:406. doi: 10.1186/1471-2164-10-406 (PMC2741493; doi:10.1186/1471-2164-10-406)
Supplement: Additional file 5 — Affymetrix quality controls of the microarrays. Microarray quality controls according to the supplier. [file 1471-2164-10-406-S5.pdf]

Affymetrix quality controls

Our arrays were submitted to the quality controls established by Affymetrix [1]. These results were obtained through the examination of the .dat and .rpt files, which were done by the software provided by Affymetrix MAS 5.0.

1.- Probe Array Image Inspection

A general visual inspection of the different arrays (.dat files) was done without observing any evident problems (scratches, saturation areas, etc.).

2.- B2 Oligo Performance

The pattern of intensities (border and corner pattern and the array name) done by B2 Oligo was observed clearly in the arrays.

3.- Average Background and Noise Values

The Average Background and Noise (RawQ) was similar among the arrays, ranged between 2.13 and 3.15 and with a median of 2.54.

4.- Hybridization Controls: bioB, bioC, bioD and cre.

All hybridization controls were called “Present” and bioB, bioC, bioD and cre have respectively increasing signal values.

5.- Internal Control Genes: GAPDH and β-actin.

The ratio of the 3’ to the 5’ GAPDH and β-actin probe set was always no more than 3. GAPDH: range between 1.04 and 1.62 with a median of 1.435. β-actin: range between 0.94 and 1.93 with a median of 1.315.

6.- Percent Present

The percentage of genes called Present remained consistent across the arrays, being into the range of 29.2 and 34.9 with a median of 31.9.

7.- Scaling and Normalization Factors

Applying a global scaling with a Target Intensity value of 150 the scaling factor was similar among all the arrays, ranged between 1.257 and 2.705 and with a median of 2.090.

Therefore, we could conclude that arrays had passed all the Affymetrix quality controls.

1. Affymetrix, Inc. “GeneChip® Expression Analysis Data Analysis Fundamentals”. Available from https://www.affymetrix.com/support/downloads/manuals/data_analysis_fundamentals_manual.pdf, accessed 16 January 2008.
