# Supplementary material for: Molecular mechanisms of tungstate-induced pancreatic plasticity: a transcriptomics approach
Source: BMC Genomics. 2009 Aug 28;10:406. doi: 10.1186/1471-2164-10-406 (PMC2741493; doi:10.1186/1471-2164-10-406)
Supplement: Additional file 6 — Statistical quality controls of the microarrays. Microarray quality controls using different packages from Bioconductor. [file 1471-2164-10-406-S6.pdf]

Statistical Quality Control

Histogram and box plot analysis of the raw data were performed with Affy package [1] [see Additional file 7 A and B], observing that all the arrays had a similar range of intensities and there was not signal saturation at the highest intensities. By plotting the histograms and the boxplots of the background adjusted, normalized and summarized raw data by Robust Multi-array Analysis (RMA) [2] [see Additional file 7 C and D], we could conclude that this transformation overcame the subtle differences between the arrays.

Furthermore, the arrays passed the outlier detection algorithm described by Li And Wong [3], which was applied when the model-based expression measures were calculated by the PM only and the PM/MM model.

Moreover, we fitted to the raw data a robust linear model by iteratively reweighted least squares, with the parameters probes and samples. Chip image plots of the weights from the robust linear model fit were drawn (data not show), without detecting any evident problem. Additionally, boxplots of the normalized unscaled standard errors (NUSE boxplot, [see Additional file 8 A]) and boxplots of the distribution of the relative logarithmic expressions (RLE boxplot, [see Additional file 8 B and C]) were plotted, observing a similar tendency in all the arrays (the boxes were in a similar range). These observations were done using the AffyPLM package [4].

Therefore, we concluded that there wasn't any outlier among the arrays.

Versions used

Affy package (version 1.6.7) and AffyPLM package (version 1.3.3) from Bioconductor [5] were performed on R language [6,7] (version 2.1.0).

The outlier detection algorithm described by Li and Wong were performed with dChip (version Jun 2 2005).

All the scripts are available under request.

1. Gautier L, Cope L, Bolstad BM, Irizarry RA. affy--analysis of Affymetrix GeneChip data at the probe level. *Bioinformatics* 2004; **20**:307-15.
2. Irizarry RA, Bolstad BM, Collin F, Cope LM, Hobbs B, Speed TP. Summaries of Affymetrix GeneChip probe level data. *Nucleic Acids Res* 2003; **31**:e15.

3. Li C, Wong WH. Model-based analysis of oligonucleotide arrays: expression index computation and outlier detection. *Proc Natl Acad Sci USA* 2001; **98**:31-6. Available from http://www.dchip.org, accessed 16 January 2008.
4. Bolstad, BM. *Low level analysis of high-density oligonucleotide array data: Background, normalization and summarization. Dissertation.* University of California, Berkeley; 2004.
5. Gentleman RC, Carey VJ, Bates DM, Bolstad B, Dettling M, Dudoit S, *et al.* Bioconductor: open software development for computational biology and bioinformatics. *Genome Biol.* 2004; **5**: R80. Available from http://www.bioconductor.org, accessed 16 January 2008.
6. Ihaka R and Gentleman R. R: A language for data analysis and graphics. *Journal of computational and graphical statistics* 1996; **3**: e15.
7. R: Development core team. *R: A language and environment for statistical computing.* R foundation for statistical computing. Viena, Austria; 2004. Available from http://www.r-project.org, accessed 16 January 2008.
