# Supplementary material for: Molecular mechanisms of tungstate-induced pancreatic plasticity: a transcriptomics approach
Source: BMC Genomics. 2009 Aug 28;10:406. doi: 10.1186/1471-2164-10-406 (PMC2741493; doi:10.1186/1471-2164-10-406)
Supplement: Additional file 9 — Microarray analysis. Explanation of the microarray analysis to obtain the differential expressed genes from each experimental group. [file 1471-2164-10-406-S9.pdf]

Microarray analysis

Background adjustment, normalization and data summarization were performed by evaluation of .cel files by Robust Multi-array Analysis (RMA) [1] using the Affy package [2]. Processed data was analyzed with the LIMMA package [3]. The selection of differentially expressed genes between conditions was based on a linear model $Y_{ijkg} = \mu_g + Treatment_{ig} + Diabetes_{jg} + (Diabetes\ and\ Treatment)_{ijg} + \varepsilon_{ijkg}$ with empirical Bayes moderation of the variance estimates following the methodology developed by Smyth [3]. The method extends traditional linear model analysis using empirical Bayes methods to combine information from the whole array and every individual gene in order to obtain improved error estimates. This method also provides the "B-statistic" which is defined as the logarithm of the posterior odds that a gene is differentially expressed vs that it is not. This statistic has been used to select genes by calling differentially expressed those genes whose B-value is greater than zero, that is, those genes where it is more likely that they are differentially expressed that they are not. With the differentially expressed genes, the genes and the samples were clustered and represented in a heat diagram with the dChip software [4].

1. Irizarry RA, Hobbs B, Collin F, Beazer-Barclay YD, Antonellis KJ, Scherf U, *et al.* Exploration, normalization, and summaries of high density oligonucleotide array probe level data. *Biostatistics* 2003; **4**: 249-64.
2. Gautier L, Cope L, Bolstad BM, Irizarry RA. affy--analysis of Affymetrix GeneChip data at the probe level. *Bioinformatics* 2004; **20**: 307-15.
3. Smyth, G. K. Linear models and empirical Bayes methods for assessing diferential expression in microarray experiments. *Statistical Applications in Genetics and Molecular Biology* 2004; **3**, No. 1, Article 3;
4. Li C, Wong WH. Model-based analysis of oligonucleotide arrays: expression index computation and outlier detection. *Proc Natl Acad Sci USA*. 2001; **98**: 31-6. [http://www.dchip.org]
