# Supplementary material for: Molecular mechanisms of tungstate-induced pancreatic plasticity: a transcriptomics approach
Source: BMC Genomics. 2009 Aug 28;10:406. doi: 10.1186/1471-2164-10-406 (PMC2741493; doi:10.1186/1471-2164-10-406)
Supplement: Additional file 10 — Primers used in the Real Time PCR. Description of the primers used in the Real Time PCR. [file 1471-2164-10-406-S10.pdf]

Primers used in the Real Time PCR.

Tbp (NM_001004198)
Forward: tgcacaggagccaagagtga
Reverse: agcccagcttctgcacaact

Tgfb3 (NM_013174)
Forward: caggcccttgcccttacc
Reverse: tcagggtgttgtatagtccaagca

Fgf13 (NM_053428)
Forward: tctcccgatccggaagtg
Reverse: ggatttgcctccattcagtacac

Xbp1 (NM_001004210)
Forward: tcctgggaggacacttttgc
Reverse: tggtgggtggctttagacact

Usag-1 (NM_153737)
Forward: agcccggtggcattttc
Reverse: gctggcattccactccaaga

Tspan8 (NM_133526)
Forward: tgcagttgggtccatcatca
Reverse: agcatgcagcgactttctttc

Sel1h (NM_177933)
Forward: ttgatgtagggctctggatggt
Reverse: tctaagcagcttctgggattcaac

Rkip (NM_017236)
Forward: acttcctggtggtcaacatgaa
Reverse: tccggagcccacgtattc

Insulin2 (NM_019130)
Forward: ttgtggttctcacttggtggaa
Reverse: cacttgtgggtcctccacttc

Amylase (NM_031502)
Forward: cattttccaagaggtcattgatctt
Reverse: tcacgcggccatttcc

Nupr1 (NM_053611)
Forward: gcctggcgctgagacaga
Reverse: ccaaggtcctgtatccattgct
